# Ag Nanoparticles and Rod-Shaped AgCl Decorated Porous PEDOT as a Bifunctional Material for Hydrogen Evolution Catalyst and Supercapacitor Electrode

**DOI:** 10.3390/molecules28248063

**Published:** 2023-12-13

**Authors:** Chunyong Zhang, Haoyu Wang, Li Shu, Zhe Li, Jirong Bai, Yinpin Wen, Lin Zhu, Yin Geng, Hengfei Qin

**Affiliations:** 1 School of Chemistry and Environmental Engineering, Jiangsu University of Technology, Changzhou 213001, China; zhangcy@jsut.edu.cn (C.Z.); 2021657055@smail.jsut.edu.cn (H.W.); 2021657032@smail.jsut.edu.cn (Z.L.); 2022657046@smail.jsut.edu.cn (L.Z.); 15152399109@163.com (Y.G.); jlgqinhf@jsut.edu.cn (H.Q.); 2 Jiangsu Key Laboratory of Precious Metal Chemistry and Technology, Jiangsu University of Technology, Changzhou 213001, China; shuli@just.edu.cn (L.S.); wenyp@jsut.edu.cn (Y.W.); 3 Research Center of Secondary Resources and Environment, School of Chemical Engineering and Materials, Changzhou Institute of Technology, Changzhou 213022, China

**Keywords:** electrocatalyst, hydrogen evolution reaction, supercapacitor electrode

## Abstract

PEDOT-Ag/AgCl is a highly promising material with dual functions of hydrogen evolution reaction (HER) and supercapacitors. In this study, a simple low-temperature stirring and light irradiation method was used to synthesize PEDOT-Ag/AgCl on the surface. Then, PEDOT-Ag/AgCl was analyzed using X-ray diffraction, scanning electron microscopy, X-ray photoelectron spectroscopy, and transmission electron microscopy. PEDOT-Ag/AgCl reacted in 1 M KOH alkaline electrolyte with an overpotential of 157 mV at 20 mA·cm^−2^ and a Tafel slope of 66.95 mv·dec^−1^. Owing to the synergistic effect of PEDOT and Ag/AgCl, this material had a small resistance (1.7 Ω) and a large specific capacitance (978 F·g^−1^ at current density of 0.5 A·g^−1^). The synthesis method can prepare nanostructured PEDOT with uniformly-distributed Ag nanoparticles and rod-shaped AgCl on the surface, which can be used as both HER electrocatalysts and supercapacitor electrodes.

## 1. Introduction

In the current society, human scientific and technological development is limited by the energy crisis and pernicious environmental problems. The existing rare fossil fuel environment will thus accelerate energy transition from fossil fuels to clean energy sources, such as hydrogen, solar energy, and bioenergy. The most direct and simple method for industrial production of pure hydrogen is electrocatalytic water splitting [1,2,3,4,5]. During the electrolysis of water, hydrogen evolution reactions (HER) and oxygen evolution reactions (OER) take place at different poles [6,7,8,9]. The efficiency of water splitting depends on the kinetics of HER and OER [10,11]. Although precious metals (e.g., Pt, Ag, Ir, Ru) are expensive and rare, their use in small quantities can completely replace transition metals (e.g., Fe, Ni, Co, Mn) in large quantities, and avoid the waste of resources [12,13,14,15,16].

Countries are paying growing attention to the development of green energy applications. New high-performance energy storage devices, such as sodium plasma batteries and supercapacitors, are becoming central to research breakthroughs. Supercapacitors can be categorized into two types: electrical double-layer capacitors (EDLCs) and pseudocapacitors. EDLCs can adsorb ions onto the surface of an electrode, such as activated carbon immersed in an electrolyte, thus forming electric double layers (EDLs) to accumulate energy electrostatically [17]. On the flip side, a series of very rapid reversible Faraday redox, electrosorption, and embedding processes will occur on electrode surfaces (electrically conducting polymers (ECPs) [18,19,20,21]. The capacity of pseudocapacitance depends on the surface area, material, and structure of the electrode [22]. Pseudocapacitance has 10–100 times larger capacity than double-layer capacitance at the same surface area [23]. ECPs as high-performance capacitor materials are represented by polymers, such as polypyrrole (PPy), poly(3,4-ethylenedioxythiphene) (PEDOT), and other derivatives [24]. Because of its superior electroactivity, high stability, and low impedance, PEDOT has attracted many researchers [25,26,27,28].

Recently, various studies have been carried out to synthesize nano-PEDOT using simple polymerization methods. Murugesan et al. used a simple hydrothermal method to polymerize PEDOT onto a 3D carbon fiber cloth to ensure the material retained 86% capacitance after 12,000 electrochemical cycles [29]. Yan et al. prepared PEDOT:PSS/MnO_2_/PEDOT via a two-step method to achieve an enhanced micro-supercapacitor, the capacitance of which reached 391.36 F cm^3^ at current density of 3.75 A·cm^3^ [30]. Similarly, doping of precious metals is the target of many studies. Chen et al. doped Ru into NiFe-LDH to effectively reduce the Volmer reaction barrier, and the overpotential of NiFeRu-LDH in a 1 M KOH alkaline electrolyte at the current density of 10 mA·cm^−2^ was only 29 mV [31]. Nazanin et al. synthesized an Ag/Cu electrocatalyst on CPE, which improved HER long-term performance by reducing the voltage and increasing the circuit density [32]. Liu et al. doped Pd into NiFe alloy, which resulted in a Tafel voltage of only 69.4 mV·dec^−1^ [33]. Among numerous precious metals, Ag is selected as a dopant owing to its relatively low price, high conductivity, regulation of electronic structure, and improved adsorption [34,35]. When we move into an era of conserving energy and reducing pollution, a dual-function electrode material is needed for HER and supercapacitors And, can not only greatly reduce costs and resources but also facilitate industrial production.

Therefore, the objective of this study is to design nanostructured PEDOT, a high-performance bifunctional electrode material, which has a huge capacitance and low electrical impedance for supercapacitors. Ag/AgCl can further reduce the electrical impedance of electrode materials and provides the active site required for HER. Thus, a simple, efficient and safe two-step process based on low-temperature stirring and UV light restoration was used to synthesize Ag/AgCl onto the surface of PEDOT nanostructures. In contrast to the hydrothermal synthesis methods described in other literature, this method only required stirring in a 60 °C water bath to synthesize PEDOT nanostructures. Then, PEDOT-Ag/AgCl was synthesized via stirring and UV light restoration at room temperature. Structurally, Ag coordinated to the S of PEDOT, while AgCl grew directly on the surface of PEDOT. Furthermore, all samples were tested in a 1 M KOH alkaline electrolyte. The structures and physicochemical properties were characterized using analytical instruments. The applicability of electrode materials in HER and supercapacitors was monitored by cyclic voltammetry (CV), electrochemical impedance spectroscopy (EIS), galvanostatic charge/discharge (GCD), linear sweep voltammetry (LSV), and Tafel methods.

## 2. Results and Discussion

### 2.1. Synthesis and Characterisation

**Figure 1:**
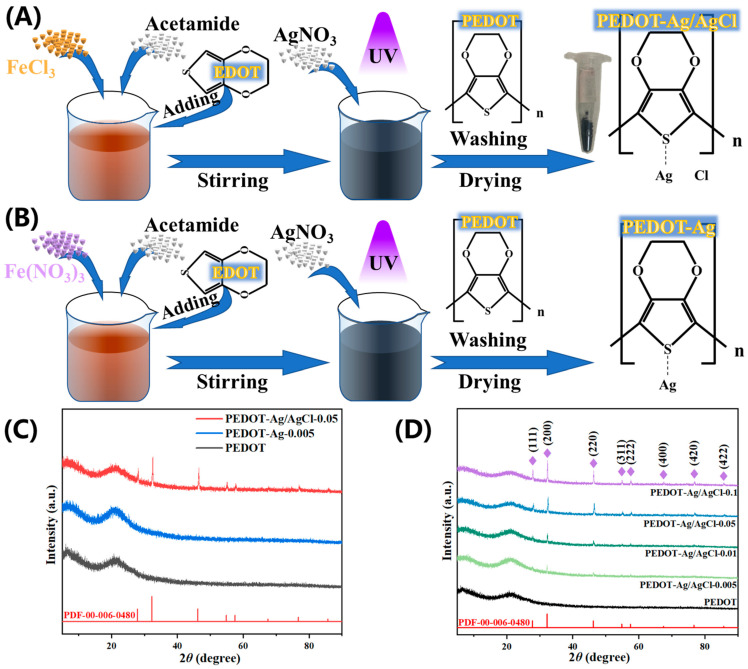
Schematic illustration of the synthesis process of (**A**) PEDOT-Ag/AgCl and (**B**) PEDOT-Ag, (**C**) XRD patterns of the PEDOT, PEDOT-Ag-0.005, and PEDOT-Ag/AgCl-0.05, (**D**) XRD patterns of the PEDOT-Ag/AgCl.

The synthesis of PEDOT-Ag/AgCl is schematically illustrated in Figure 1A. First, EDOT was added to the EDS liquid system for polymerization to obtain blue-black PEDOT. Second, AgNO_3_ was dripped into the PEDOT mixture to form PEDOT-Ag/AgCl (FeCl_3_ + AgNO_3_ → Fe(NO_3_)_3_ + AgCl), which was obtained due to the participation of some silver ions in the single displacement reaction. Under the same synthesis steps, FeCl_3_·6H_2_O was replaced with Fe(NO_3_)_3_·9H_2_O to obtain PEDOT-Ag. The structural diagrams of PEDOT and PEDOT-Ag were shown in Figure 1B, while AgCl growing on the surface of PEDOT-Ag was named PEDOT-Ag/AgCl [36]. All samples exhibited a characteristic diffraction peak in 15–25° resembling PEDOT (Figure 1C,D). This amorphous peak was determined for PEDOT [37,38,39]. X-ray diffraction (XRD) shows eight diffraction peaks of PEDOT-Ag/AgCl (Figure 1D), corresponding to (111), (200), (220), (311), (222), (400), (420), and (422) crystal planes, respectively. In this test, the primary diffraction peaks of PEDOT-Ag/AgCl appear at 2*θ* = 27.88°, 32.36°, 46.32°, 54.91°, 57.58°, 67.56°, 76.82°, 85.81°, which well-match with the peaks of AgCl (PDF#00-006-0480 for AgCl) [40]. The diffraction peaks of AgCl become broader and stronger with the increase in AgNO_3_ content. When the molar ratio of S:Ag reached 1:0.1, the peak intensity was the highest. Moreover, the intensity of the diffraction peaks conforms to the experimental rules. In combination with Figure 1B,C, no obvious diffraction peaks of Ag nanoparticles can be found, which is due to the low doping of Ag in the samples. Other literature also shows no diffraction peak of Ag [41,42,43].

**Figure 2:**
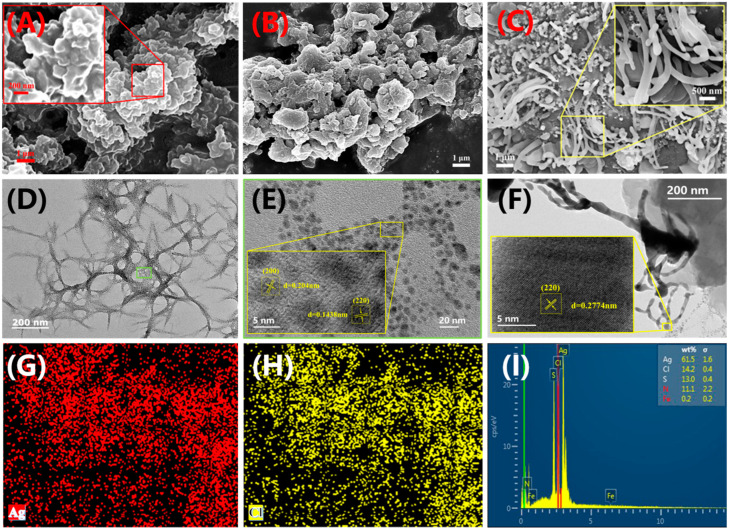
SEM images of (**A**) PEDOT, (**B**) PEDOT-Ag-0.005, and (**C**) PEDOT-Ag/AgCl-0.05. (**D**–**F**) TEM images of PEDOT-Ag/AgCl-0.05. (**G**–**I**) EDS element mappings of PEDOT-Ag/AgCl-0.05.

PEDOT exists in a stacked flaky form (Figure 2A). The scanning electron microscopy (SEM) image of PEDOT-Ag-0.005 is shown in Figure 2B. In comparison with PEDOT (Figure 2A), although the uneven surface morphology is similar, PEDOT-Ag-0.005 in Figure 2B exhibits a morphological feature of particle stacking due to the presence of Ag nanoparticles [36]. Figure 2C shows the SEM image of PEDOT-Ag/AgCl-0.05. Compared with Figure 2A,B, PEDOT-Ag/AgCl-0.05 has numerous rod-shaped AgCl morphological structures in size of 200–300 nm on the surface of PEDOT-Ag. After dispersion of PEDOT-Ag/AgCl-0.05, PEDOT-Ag shows an interconnected needle-like and porous morphological structure on the TEM image in Figure 2D. At this time, the rod-shaped AgCl morphological structure on the surface of PEDOT-Ag/AgCl fell off due to ultrasonic dispersion (Figure 2F). On Figure 2E zoomed at a scale of 5 nm from Figure 2D, the lattices of Ag nanoparticles are evident [44]. The lattice fringes of the Ag (200) and (220) layers are displayed in Figure 2E, and the lattice spacing is d = 0.204 and 0.1438 nm, respectively, which are very similar to the properties of Ag nanoparticles. Figure 2F shows the rod-shaped structure of AgCl, and the lattices are clearly seen at the scale of 5 nm. The lattice spacing of AgCl is 0.2774 nm, which is close to the characteristic of AgCl (220) layer [41,43,45]. In Figure 2G,H, AgCl is uniformly distributed on the surface of PEDOT-Ag. The mass percentage ratio of Ag and Cl is 61.5:14.2 (Figure 2I), which proves the co-existence of Ag nanoparticles and AgCl. When PEDOT-Ag/AgCl-0.05 was synthesized using FeCl_3_·6H_2_O, some silver ions and chloride ions combined to form AgCl after the addition of AgNO_3_, but the iron ions were almost completely dissolved in ethanol and washed.

**Figure 3:**
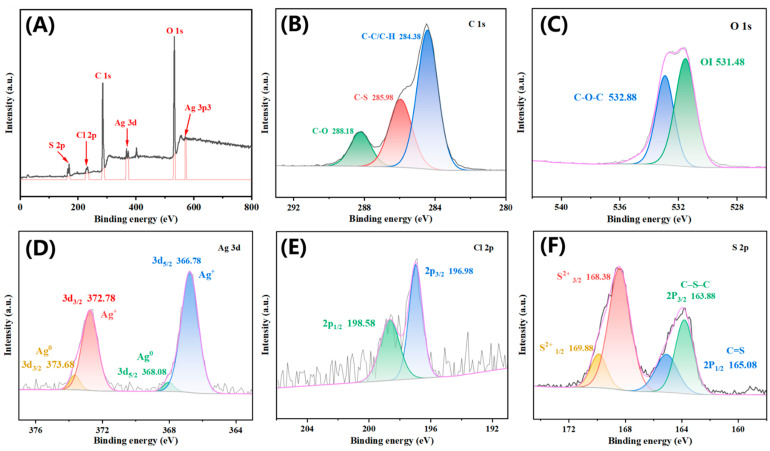
XPS of PEDOT-Ag/AgCl-0.05 (**A**) XPS survey, (**B**) C 1s, (**C**) O1s (**D**) Ag 3d, (**E**) Cl 2p, and (**F**) S 2p of high-resolution spectra.

The X-ray photoelectron spectroscopy (XPS) spectrum of PEDOT-Ag/AgCl-0.05 reveals the presence of carbon (C), oxygen (O), silver (Ag), chlorine (Cl), and sulfur (S) (Figure 3A). The C 1s spectrum in Figure 3B displays three peaks at 284.38, 285.98, and 288.18 eV, corresponding to C-C/C-H, C-S, and C-O, respectively [46,47]. The peaks in O 1s are located at 531.48 and 532.88 eV, corresponding to lattice oxygen (OI) and C-O-C, respectively [48,49]. There are two typical peaks Ag 3d_5/2_ and Ag 3d_3/2_ in Figure 3D of Ag 3d. The peaks at 367.5 and 373.38 eV can be attributed to Ag^+^ in AgCl, while the peaks at 368.18 and 374.18 eV belong to Ag^0^. The peak at 573.08 eV in Figure 3A is characteristic of Ag 3p3 [49]. The peaks at 196.98 and 198.58 eV in Figure 3E are assigned to Cl 2p_3/2_ and Cl 2p_1/2_, respectively. These peaks originate from Cl^-^ in AgCl [50]. The S 2p spectrum in Figure 3F shows peaks at 163.88 and 165.08 eV that are ascribed to S 2p_3/2_ (C-S-C) and S 2p_1/2_ (C=S), respectively [51]. The BEs at 168.38 and 169.88 eV with prominent doublet correspond to S^+^ 2p, indicating partial S atoms in PEDOT may exist in an oxidized form or combine with Ag [52]. In summary, this synthesis method can enable the presence of Ag and AgCl on PEDOT-Ag/AgCl-0.05.

### 2.2. Electrochemical Properties

**Figure 4:**
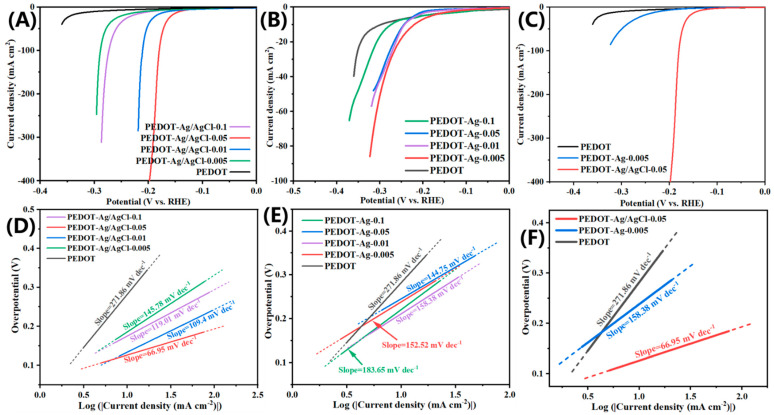
LSV curves for (**A**) PEDOT-Ag/AgCl, (**B**) PEDOT-Ag, (**C**) PEDOT-Ag/AgCl-0.05, PEDOT-Ag-0.005, and PEDOT. Tafel plots for (**D**) PEDOT-Ag/AgCl, (**E**) PEDOT-Ag, (**F**) PEDOT-Ag/AgCl-0.05, PEDOT-Ag-0.005, and PEDOT.

All electrochemical tests were conducted in 1 M KOH alkaline electrolyte [53]. The overpotentials of PEDOT-Ag/AgCl-0.1, PEDOT-Ag/AgCl-0.05, PEDOT-Ag/AgCl-0.01, PEDOT-Ag/AgCl-0.005, and PEDOT reached 231, 157, 187, 249, and 344 mV, respectively, at the current density of 20 mA·cm^−2^ and scanning rate of 5 mV·s^−1^ (Figure 4A). The order of overpotentials is PEDOT-Ag/AgCl-0.05 < PEDOT-Ag/AgCl-0.01 < PEDOT-Ag/AgCl-0.1 < PEDOT-Ag/AgCl-0.005 < PEDOT (Figure 4B). Under the same conditions, the order of overpotentials of PEDOT-Ag is PEDOT-Ag-0.005 < PEDOT-Ag-0.01 < PEDOT-Ag-0.05 < PEDOT-Ag/AgCl-0.01 < PEDOT. The PEDOT had a maximum overpotential of 343 mV at 20 mA·cm^−2^, and the minimum overpotential was 255 mV in PEDOT-Ag-0.005. At this time, the overpotential of PEDOT-Ag/AgCl-0.05 was 186 mV lower than that of PEDOT and 98 mV lower than that of PEDOT-Ag-0.005 (Figure 4C). In comparison, under the synergetic action of Ag/AgCl, not only the electrode had a low overpotential, greatly reducing the energy required for HER, but also its current density exceeded 400 mA·cm^−2^.

The Tafel slope is an important parameter for evaluating the performance of the hydrogen evolution reaction (HER) and determining the rate-limiting step. In an alkaline solution, there are three reaction processes for HER as follows:

The Volmer reaction involves the reduction of water to adsorbed hydrogen and hydroxide ions:

H_2_O + e^−^ → H_ads_ + OH^−^

The Heyrovsky reaction involves the combination of adsorbed hydrogen with water to produce molecular hydrogen and hydroxide ions:

H_ads_ + H_2_O + e^−^ → H_2_ + OH^−^

The Tafel reaction represents the reaction between two adsorbed hydrogen species to form molecular hydrogen:

H_ads_ + H_ads_ → H_2_

For the Tafel plot’s linear section, the equation *η* = *a* + *b* × log *j* is used, with *b* representing the Tafel slope and *a* denoting the material’s inherent constant. Combining the Tafel slope images (Figure 4D–F) of PEDOT-Ag and PEDOT-Ag/AgCl, it can be seen the five samples’ Tafel slopes of PEDOT-Ag are all higher than 120 mV·dec^−1^. The Tafel slopes of PEDOT-Ag-0.1, PEDOT-Ag-0.05, PEDOT-Ag-0.01, and PEDOT-Ag-0.005 are 183.65 mV·dec^−1^, 144.75 mV·dec^−1^, 158.38 mV·dec^−1^, and 152.52 mV·dec^−1^, respectively. The order of Tafel slopes was as follows: PEDOT-Ag/AgCl-0.05 (66.95 mV·dec^−1^) < PEDOT-Ag/AgCl-0.01 (109.4 mV·dec^−1^) < PEDOT-Ag/AgCl-0.1 (119.01 mV·dec^−1^) < PEDOT-Ag/AgCl-0.005 (145.78 mV·dec^−1^) < PEDOT (271.86 mV·dec^−1^). The smallest Tafel slope was PEDOT-Ag/AgCl-0.05 (66.95 mV·dec^−1^), which indicated PEDOT-Ag/AgCl-0.05 exhibited higher catalytic activity than other examples. PEDOT-Ag/AgCl-0.05 had better performance than most PEDOT catalysts reported to date (Table 1). Compared with other catalysts in alkaline solution at a current density of 10 mA cm^−2^, the overpotential of CFP/PEDOT/Ru-Pi, Co_0.5_Zn_0.5_MoO_4_, and NiSSe are 350, 201, and 154 mV, respectively [54,55,56]. As shown in the Figure 4A–F, PEDOT-Ag/AgCl-0.05 has a smaller overpotential (157 mV) and a lower Tafel slope (66.95 mV·dec^−1^) at a current density of 20 mA cm^−2^. In this way, the Tafel slope of the catalysts in alkaline solution is much larger than that of the present work. Compared with other Ag based catalysts, even though Ag(10)WS_2_, Ag-Pd nanoalloy, and C_3_N_4_/AgPd are in acidic solution [57,58,59], the overpotential and Tafel are still higher than PEDOT-Ag/AgCl-0.05, which indicates that based on the structure of PEDOT-Ag/AgCl-0.05. In conclusion, PEDOT-Ag/AgCl-0.05 has excellent catalytic activity and good hydrogen evolution reaction performance.

**Figure 5:**
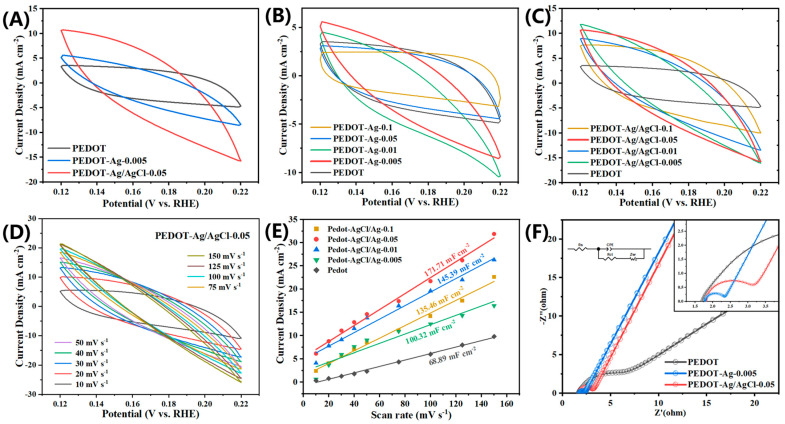
The electrochemical double-layer capacitances of (**A**) PEDOT, PEDOT-Ag-0.005, and PEDOT-Ag/AgCl-0.05, (**B**) PEDOT-Ag, (**C**) PEDOT-Ag/AgCl at a scan rate of 50 mV·s^−1^, and (**D**) PEDOT-Ag/AgCl-0.05 at various scan rates (10, 20, 30, 40, 50, 75, 100, 125, and 150 mV·s^−1^), (**E**) capacitive current density versus scan rate curves for ECSA measurements for PEDOT-Ag/AgCl-0.05, (**F**) EIS plots of PEDOT, PEDOT-Ag-0.005, and PEDOT-Ag/AgCl-0.05 from 10 ^−2^ to 10^5^ Hz.

In the CV, the cells of the examples are calculated as *C_cell_* = (∫*i*d*ʋ*)/(2*Aʋ*Δ*V*), where *i* is the current in the CV curve, *A* (cm^2^) is the working area, *ʋ* is the voltage scanning rate, Δ*V* is the voltage window, and (∫*i*d*ʋ*) is the area of the CV curve [29]. Figure 5B,C shows the CV curves of PEDOT-Ag and PEDOT-Ag/AgCl at a scanning speed of 50 mV·s^−1^, respectively. Clearly, the curve integral areas of PEDOT-Ag-0.005 and PEDOT-Ag/AgCl-0.05 are the largest. The cells of PEDOT-Ag-0.1, PEDOT-Ag-0.05, PEDOT-Ag-0.01, PEDOT-Ag-0.005, and PEDOT are 390.3, 428.9, 467.2, 506.9, and 485.1 F·g^−1^, respectively. The cells of PEDOT-Ag/AgCl-0.1, PEDOT-Ag/AgCl-0.05, PEDOT-Ag/AgCl-0.01, PEDOT-Ag/AgCl-0.005, and PEDOT are 887.7, 1003, 784, 733.1, and 495.1 F·g^−1^, respectively. As shown in Figure 5A, the CV curve area of PEDOT-Ag/AgCl-0.05 is larger than that of PEDOT or PEDOT-Ag-0.005. It can be determined PEDOT-Ag/AgCl-0.05 has a large specific capacitance. At the scanning rate of 10–150 mV·s^−1^, the width of the CV curve significantly increases (Figure 5D), which conforms to the electrochemical law [60]. The calculation results of ECSA are shown in Figure 5E. ECSA of PEDOT-Ag/AgCl-0.05 is 171.71 mF·cm^−2^, which is 2.5 times higher than that of pristine PEDOT [61,62]. Combined with the CV curve, it is clear with the increase in Ag content, the specific capacitance is continuously increased to reach its maximum when the ratio of S:Ag is 1:0.05 (PEDOT-Ag/AgCl-0.05).

Figure 5F shows the Nyquist plots of the PEDOT, PEDOT-Ag-0.005, and PEDOT-Ag/AgCl-0.05 electrodes. A Nyquist plot is composed of a semicircular part in the high-frequency range and a linear part in the low-frequency range. The semicircular part corresponds to the electron transfer restriction process, and a larger part means a greater resistance. The linear part corresponds to the diffusion restriction process [46,54,63,64]. The values of Rs and Rct have been shown in Table 2. In a 1 mol NaOH solution, the Rs values of PEDOT, PEDOT-Ag-0.005, and PEDOT-Ag/AgCl-0.05 are 1.69, 1.80, and 1.78 Ω, respectively. Combined with Figure 5F, the resistance of PEDOT-Ag-0.005 (Rct = 0.6 Ω) and PEDOT-Ag/AgCl-0.05 (Rct = 1.7 Ω) is much smaller than that of PEDOT (Rct = 5.5 Ω) in the high-frequency region, which accelerates the electron transfer rate. Due to the presence of AgCl, the resistance of PEDOT-Ag/AgCl-0.05 is slightly higher than that of PEDOT-Ag-0.005. In the low-frequency region, the angles of the diffusion range of PEDOT-Ag-0.005 and PEDOT-Ag/AgCl-0.05 are both larger than 45°, which prove the good HER performances.

**Figure 6:**
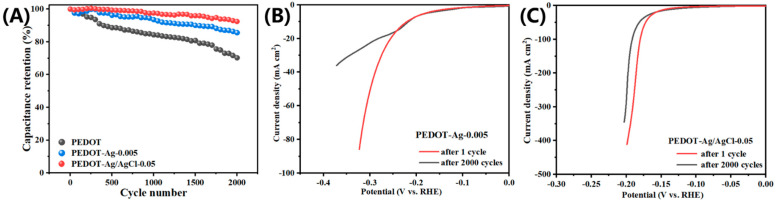
(**A**) The cyclic performance of PEDOT, PEDOT-Ag-0.005, and PEDOT-Ag/AgCl-0.05, HER polarization curves of (**B**) PEDOT-Ag-0.005 and (**C**) PEDOT-Ag/AgCl-0.05 before and after 2000 cycles CV scans.

Figure 6A shows the cyclic performances of the PEDOT, PEDOT-Ag-0.005, and PEDOT-Ag/AgCl-0.05 electrodes at 1.0 A·g^−1^. The PEDOT, PEDOT-Ag-0.005, and PEDOT-Ag/AgCl-0.05 electrodes can maintain 70.29%, 85.60%, and 92.42% of the initial capacities after 2000 cycles, respectively, while PEDOT-Ag/AgCl-0.05 outperforms the other two materials, which reflects its excellent electrochemical performance [29]. Moreover, the overpotential of PEDOT-Ag-0.005 changes significantly when the current density exceeds 20 mA·cm^−2^, while PEDOT-Ag/AgCl-0.05 shows no significant change after 2000 CV cycles, confirming its favorable endurance (Figure 6B,C) [65].

**Figure 7:**
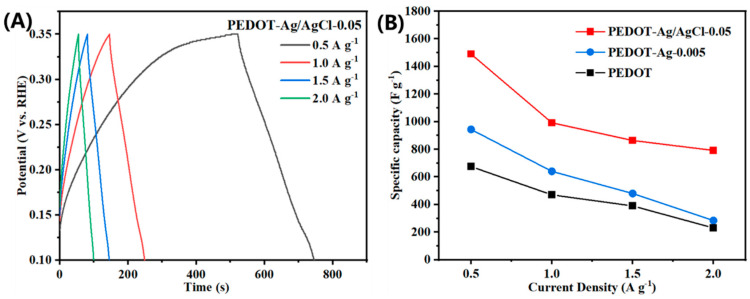
(**A**) Galvanostatic curves of PEDOT-Ag/AgCl-0.05 at various current densities (0.5, 1.0, 1.5, and 2.0 A g^−1^). (**B**) Capacitance retention property of PEDOT, PEDOT-Ag-0.005, and PEDOT-Ag/AgCl-0.05 at current densities from 0.5 to 2.0 A g^−1^.

GCD measurements of PEDOT-Ag/AgCl-0.05 were conducted at various current densities (0.5, 1.0, 1.5, and 2.0 A·g^−1^) from 0.1 to 0.35 V. The charge curve of PEDOT-Ag/AgCl-0.05 is linear and symmetrical to the discharge curve (Figure 7A). In addition, the charge and discharge voltages of PEDOT-Ag/AgCl-0.05 are non-linearly related with time, representing typical pseudocapacitive characteristics. At a current density close to 1.0 A·g^−1^, the specific capacitance of the PEDOT-Ag/AgCl-0.05 electrode was calculated as per *C_cell_* = *i*Δ*t*/Δ*V* (where *i* is the constant discharge current density, Δ*t* is the discharging duration, and Δ*V* is the potential window) to be 978 F·g^−1^, and its charge-discharge curve exhibits a longer discharge plateau [30,66,67]. The specific capacitance of the PEDOT, PEDOT-Ag-0.005, and PEDOT-Ag/AgCl-0.05 electrodes was tested at four current densities from 0.5 to 2.0 A·g^−1^. Although the specific capacitance of PEDOT-Ag/AgCl-0.05 decreased, it was still around 1000 F·g^−1^ at 2.0 A·g^−1^, which is much larger than those of PEDOT and PEDOT-Ag-0.005. In summary, PEDOT-Ag/AgCl-0.05 shows good electrochemical performance, which may be because the increased contact between the electrode and the electrolyte, thanks to the rod-like AgCl and the Ag nanoparticles, stores more active sites and thus endows PEDOT-Ag/AgCl-0.05 with good capacitive performance.

## 3. Experimental Section

### 3.1. Reagents and Materials

Acetamide (C_2_H_5_NO, AR, 99%, CAS: 60-35-5), isopropyl alcohol (C_3_H_8_O, AR, ≥99.7%, CAS: 67-63-0), ethanol (CH_3_CH_2_OH, AR, 99.5%, CAS: 64-17-5), potassium hydroxide (KOH, AR, 99%, CAS: 1310-58-3) poly (tetrafluoroethylene) (PTFE, (C_2_F_4_)n, CAS: 9002-84-0) and 3,4-ethylenedioxythiophene (EDOT, C_6_H_6_O_2_S, AR, 99%, CAS: 126213-50-1) were obtained from Shanghai Aladdin Biochemical Technology Co., Ltd. (Shanghai, China). Ferric chloride hexahydrate (FeCl_3_·6H_2_O, AR, 99%, CAS: 10025-77-1) and ferric nitrate nonahydrate (Fe(NO_3_)_3_·9H_2_O, AR, 99%, CAS: 7782-61-8) were commercially purchased from Sinopharm Chemical Regent Co., Ltd. (Shanghai, China). Silver nitrate (AgNO_3_, AR, 99%, CAS: 7761-88-8) were purchased from Shanghai Zhanyun Chemical Co., Ltd. (Shanghai, China). All materials and chemicals were used without further purification.

### 3.2. Preparation of PEDOT

In a typical study, the experiment constructed a liquid DES (deep eutectic solvent) system by using acetamide and FeCl_3_·6H_2_O to provide hydrogen bonds for EDOT polymerization. acetamide and FeCl_3_·6H_2_O (the molar ratio of acetamide and FeCl_3_·6H_2_O = 4:1) were stirred in an oil bath at 60 °C for 20 min to obtain a reddish-brown DES liquid [44]. By directly dripping EDOT slowly into the DES liquid under magnetic stirring at 400 rpm, a large amount of blue-black flocculent precipitates was produced. Finally, PEDOT was obtained via rinsing with ethanol and drying at 60 °C for 8 h.

### 3.3. Preparation of PEDOT-Ag and PEDOT-Ag/AgCl

In the process of the previous experiment, 7.15 mL silver nitrate solution (0.5 wt% dispersion in H_2_O) was added to stir for 20 min after the production of blue-black flocculent precipitation, and then the final product was obtained by adding methanol with a UV lamp for 20 min. The PEDOT-Ag and PEDOT-Ag/AgCl electrode materials were obtained after rinsing with ethanol and drying at 60 °C for 8 h for further analysis (Figure 1A). The molar ratios of element S:Ag were adjusted to 1:0.1, 1:0.05, 1:0.01, 1:0.005 and 1:0, corresponding to samples PEDOT-Ag/AgCl-0.1, PEDOT-Ag/AgCl-0.05, PEDOT-Ag/AgCl-0.01, PEDOT-Ag/AgCl-0.005, and PEDOT (Figure 1A). By the way, PEDOT-Ag was synthesized using the same method, only replacing FeCl_3_·6H_2_O with Fe(NO_3_)_3_·9H_2_O. In the same way, samples PEDOT-Ag was named PEDOT-Ag-0.1, PEDOT-Ag-0.05, PEDOT-Ag-0.01, and PEDOT-Ag-0.005 by the molar ratio of elements S and Ag (Figure 1B).

### 3.4. Characterization and Electrochemical Measurements

The morphological and structural properties of all examples were characterized using X-ray diffraction (XRD, PW3040/60, Philips, Tokyo, Japan), scanning electron microscopy (SEM, Sigma 300, Zeiss, Oberkochen, Germany), X-ray spectroscopy (EDS), ultra-high-resolution transmission electron microscope (TEM, JEM 2100, JEOL, Tokyo, Japan), and XPS analysis. The diffraction peaks and crystal plane structure of the samples were recorded and analyzed by XRD (Cu Ka radiation scanning angle 2*θ* = 5° to 90°, target voltage = 40 kV, current = 30 mA). The structure, size, and other characteristics of the samples PEDOT, PEDOT-Ag-0.005, and PEDOT-Ag/AgCl-0.05 were analyzed via using an Inlens of SEM. Elemental distribution and content of PEDOT-Ag/AgCl-0.05 were confirmed through EDS. Meanwhile, the ultrastructure and crystal lattice of the sample PEDOT-Ag/AgCl-0.05 were observed by TEM. The sample PEDOT-Ag/AgCl-0.05 was determined by XPS and analyzed by Avantage 5.984 software for the presence and valence states of the elements.

### 3.5. Electrochemical Measurements

All electrochemical tests were measured at an electrochemical workstation (CHI760E, Shanghai, China). PEDOT-Ag/AgCl and polytetrafluoroethylene micropowder were mixed at a mass ratio of 8:1, then they were added to an isopropyl alcohol solution. After ultrasonic vibration, they were put into the oven for a certain time to become a kind of muddy mixture. Subsequently, a certain amount of the mixture was taken by a liquid transfer gun to spread it evenly over the surface of the foam nickel (1 × 1 cm^2^) [54]. Finally, the electrode was obtained via pressing the foam nickel on the tablet press after drying at 60 °C for 8 h. Electrochemical tests were reacted in 1.0 M KOH alkaline electrolyte via a standard three-electrode system (a working electrode, a Pt sheet of counter electrode, and a saturated calomel reference electrode (RHE, E_RHE_ = E_Hg/Hg2Cl2_ + 0.0592 × pH + 0.24 V)). To this end, the Tafel slopes of electrodes were fitted from Linear sweep voltammetry (LSV) with a scan rate of 5 mV s^−1^ and verified through calculation. The double-layer capacitance (Cdl) of the electrochemical system was determined through cyclic voltammetry (CV) measurements at scan rates of 10, 20, 30, 40, 50, 75, 100, and 125 mV s^−1^ over the non-Faradaic region. In addition, electrochemical impedance spectrum (EIS) was tested under the frequency from 100 kHz to 0.1 Hz with a scan rate of 5 mV s^−1^ in the electrolyte. The sample was allowed to pass through 2000 CV cycles, and the capacity retention rate was obtained. Galvanostatic charge-discharge measurements of PEDOT-Ag/AgCl-0.05 were conducted at various current densities (0.5, 1, 1.5, and 2 A g^−1^) in a potential window of 0.1 to 0.35 V.

## 4. Conclusions

PEDOT-Ag-0.005 and PEDOT-Ag/AgCl-0.05 were prepared through two different methods, and PEDOT-Ag/AgCl-0.05 showed better HER and supercapacitor performances than PEDOT and PEDOT-Ag-0.005. The main HER and supercapacitor performances of PEDOT-Ag/AgCl-0.05 were reflected in three aspects. (1) After Ag and AgCl combined, the resistance of PEDOT was significantly reduced, which led to an improvement in electrochemical performance. (2) The presence of Ag nanoparticles and rod-shaped AgCl on the surface of PEDOT-Ag/AgCl greatly enlarges the contact area with the electrolyte, which facilitates the adsorption, diffusion, and storage of ions. (3) As a pseudocapacitor, PEDOT is significantly improved in specific capacitance after doping with Ag and loading with AgCl. In addition, the overpotential of PEDOT-Ag/AgCl-0.05 at 20 mA·cm^−2^ is 157 mV, the Tafel slope is 66.95 mV·dec^−1^, the electrical impedance is only 1.7 Ω, and the ECSA is 171.71 mF·cm^−2^. The specific capacitance reaches 978 F·g^−1^ at a current density of 0.5 A·g^−1^, and still maintains 92.42% after 2000 CV cycles, which shows the excellent performance of this dual-functional material. Therefore, this material provides a research foundation for the future development of more efficient HER catalysts and supercapacitors.

## Figures and Tables

**Table 1:** Comparative setup shows HER activities in the present work and some reports.

Catalysts	Electrolyte	*J* (mA·cm^−2^)	Overpotential (mV)	Tafel Slope (mV·dec^−1^)	Ref.
CFP/PEDOT/Ru-Pi	1.0 M PBS	10	350	-	[54]
Co_0.5_Zn_0.5_MoO_4_	1.0 M KOH	10	201	162.7	[55]
NiSSe	1.0 M KOH	10	154	125	[56]
Ag(10)WS_2_	0.5 M H_2_SO_4_	10	170	40	[57]
Ni_95_Fe_5_/CP	1.0 M KOH	20	130	95.4	[11]
Ag-Pd nanoalloy	0.5 M H_2_SO_4_	10	270	156	[58]
C_3_N_4_/AgPd	0.5 M H_2_SO_4_	10	308	120	[59]
PEDOT-Ag-0.005	1.0 M KOH	20	255	158.38	This work
PEDOT-Ag/AgCl-0.05	1.0 M KOH	20	157	66.95	This work

**Table 2:** EIS parameters of electrodes in 1.0 M KOH solution.

Catalysts	Electrolyte	Rs (Ω cm^2^)	Rct (Ω cm^2^)
PEDOT	1.0 M KOH	1.69	0.6
PEDOT-Ag-0.005	1.0 M KOH	1.80	1.7
PEDOT-Ag/AgCl-0.05	1.0 M KOH	1.78	5.5

## Data Availability

Data are contained within the article.
